# An integrated psycho-social intervention to improve self-efficacy toward TB treatment uptake and infection prevention among patients and family caregivers: a multicentric implementation research study protocol

**DOI:** 10.3389/ftubr.2026.1713844

**Published:** 2026-02-13

**Authors:** Karikalan Nagaraj, Bella Devaleenal Daniel, Chandra Suresh, Dhanalakshmi Angamuthu, Shobana Palanisamy, Muniyandi Malaisamy

**Affiliations:** 1 Department of Social and Behavioural Research, Indian Council of Medical Research-National Institute for Research in tuberculosis, Chennai, Tamil Nadu, India; 2 Department of Clinical Research, Indian Council of Medical Research-National Institute for Research in Tuberculosis, Chennai, Tamil Nadu, India; 3 Department of Health Economics, Indian Council of Medical Research-National Institute for Research in Tuberculosis, Chennai, Tamil Nadu, India

**Keywords:** implementation, infection control, psycho-social, self-efficacy, self-management, treatment adherence, tuberculosis

## Abstract

**Background:**

The individuals with tuberculosis (TB) experience a variety of barriers, needs, and challenges when receiving the treatment. Evidence on holistic patient-centered psychosocial interventions that promote self-efficacy and the ability to complete treatment-related tasks and goals is scarce in India.

**Objectives:**

To adapt an intervention aimed at strengthening the self-efficacy of individuals on TB treatment and their family caregivers, and to evaluate its effectiveness compared with the standard of care on the primary outcome of improved TB treatment self-efficacy and secondary outcomes of improved medication adherence, reduced TB-related stigma, and improved infection control practices.

**Methods:**

A multicentric hybrid Type I mixed method implementation research design will be used to adapt and evaluate the implementation feasibility and effectiveness of delivering a self-efficacy-based intervention under National Tuberculosis Elimination Program (NTEP) settings in India. A two-arm non-randomized cluster intervention design will be employed to evaluate the impact of a self-efficacy intervention, which includes individual counseling and peer group sessions (*n* = 240) in comparison to the standard of care (*n* = 240) on primary and secondary outcomes. Qualitative in-depth interviews and focus group discussions, guided by the Consolidated Framework for Implementation Research (CFIR), will be conducted to explore contextual factors influencing implementation.

**Discussion:**

The proposed study could generate evidence for a holistic and evidence-informed psycho-social intervention for individuals with TB and their family caregivers. It aims to improve their self-efficacy in overcoming TB treatment challenges and address the key psycho-social barriers comprehensively. Qualitative insights from the study are expected to guide/facilitate the pragmatic scale-up of self-efficacy-based interventions under the National TB program of India.

**Clinical trial registration:**

The study has been registered in the Clinical Trial Registry of India CTRI/2024/05/066847.

## Background

Individuals with tuberculosis (TB) experience a range of physical and psycho-social challenges (1). Active tuberculosis (TB) deteriorates both physical and mental health, while TB treatment-related difficulties include long treatment duration, medication side effects, tablet burden, poor adherence, stigma, financial limitations, and limited health system assistance, all of which contribute to treatment interruption and poor outcomes (2). Disease denial, fear of death, and perceived infection risk contribute to fear, emotional distress, and anxiety among patients and caregivers (3). At the time of diagnosis and during the early phase of treatment, patients often experience stigma, hopelessness, and even suicidal ideation. Consequently, adverse psycho-social and life experiences, such as anxiety, depression, tension, and low self-esteem, are further intensified by treatment burden, medication intolerance, physical deterioration, and other concerns encountered throughout the treatment course (4).

The mental health of individuals with TB and their caregivers is influenced by the perceived burden of treatment and concerns related to infection risk. The disease and treatment-associated challenges impose a substantial psycho-social and economic burden on family caregivers, who are themselves at risk of acquiring infection during the caregiving process. Multiple issues may potentially arise for individuals with tuberculosis (TB) and their caregivers during the treatment cascade, subsequently affecting treatment adherence and outcomes. A recent meta-analysis revealed that poor outcomes, such as mortality, loss to follow-up, and disease recurrence, are six times higher when more than 10% of doses are missed during TB therapy (5). While TB treatment and diagnosis are provided free of cost, the acceptability of TB services is limited due to the poor knowledge, attitude and practice of the community toward TB. Stigma and incorrect perceptions lead to poor health-seeking behavior, such as delayed treatment, inadequate infection prevention, and non-adherence, which contribute to the development of drug resistance (6, 7).

An effective intervention that significantly enhances the self-efficacy of individuals with TB in treatment management and infection prevention is essential for achieving the national TB elimination goals, including reducing transmission and increasing the treatment success rate. The ability of individuals with TB and their caregivers to cope with the multifaceted challenges they encounter daily largely depends on their level of self-efficacy. In India, there is a lack of evidence-based interventions aimed at enhancing/strengthening the self-efficacy of TB patients and their caregivers in the context of treatment uptake/adherence and infection prevention. Although the treatment efficacy among individuals with TB is generally low, no studies or interventions have been reported in India that specifically address treatment self-efficacy (8, 9). Currently, there is meager evidence on psycho-social interventions that are effective, acceptable, and scalable in improving treatment self-efficacy. Therefore, we propose a study to adapt and evaluate a self-efficacy-based intervention designed to support individuals with TB and their caregivers in strengthening their actions, behaviors, and self-management skills to effectively address the challenges and demands of TB disease and treatment.

## Primary objectives

1. To adapt and evaluate the self-efficacy-based intervention compared with the standard of care on the following outcomes:
  • Primary outcomes: *improved TB treatment self-efficacy*.
  • Secondary outcomes: *(i) improved medication adherence (ii) reduced TB-related stigma and (iii) improved infection control self-management practices*.
2. To evaluate the efficacy of a self-efficacy-based intervention, compared with the standard of care, on secondary outcomes among caregivers: *improved caregivers' self-efficacy, decreased TB-related stigma, and improved infection control self-management practices*.
3. To assess the acceptability level among patients, caregivers, and health care providers.

## Hypothesis/research question

A self-efficacy-based intervention will be both clinically (*d* > 0.3) and statistically (*p* < 0.05) more effective in improving treatment self-efficacy among individuals with TB, as well as enhancing treatment adherence, infection control practices, and reducing TB-related stigma compared with the standard of care.

## Overall design

A hybrid mixed-method implementation design will be employed to evaluate the implementation acceptability and preliminary efficacy of delivering a self-efficacy-based intervention by front-line healthcare staff, family caregivers, and patient champions. The intervention aims to enhance treatment self-efficacy, mental health, and self-management practices related to infection control among individuals with TB and caregivers.

## Design for primary objective 1

A self-efficacy-based intervention manual and standard operating procedures were developed by adapting the findings from formative research conducted among individuals with TB, caregivers, and front-line healthcare staff. The PRODUCES framework (Problem, Objective and Design, End-Users, Co-creators, Evaluation, and Scalability) was used to guide the design and development of this implementation research study (10).

The self-efficacy drive intervention manual and standard operating procedures (SOPs) will be further adapted to align with the workflow of government TB care delivery systems at the proposed study sites (11).

## Design for primary objective 2

A two-arm, non-randomized cluster intervention study will be conducted to achieve this objective. The study population will include

1. Adult drug-sensitive (DS) TB patients (> 18 years) who have initiated TB treatment.
2. Patients with low treatment self-efficacy (self-efficacy score of < 30 on the chronic diseases self–efficacy scale) (12).
3. Adult drug-resistant (DR)-TB patients on a shorter treatment regimen.
4. Caregivers of patients who meet criteria 1–3 above.

## Study sites

In the proposed research, individuals with tuberculosis (TB) will be enrolled in the National Tuberculosis Elimination Program (NTEP) facilities located in selected tuberculosis treatment units (TUs) across three districts in Tamil Nadu—Chennai, Kanchipuram, and Tiruvallur. Treatment initiation and follow-up will be carried out under the supervision of NTEP staff.

## Implementation

A prospective quantitative and qualitative evaluation of the proposed intervention will be conducted among individuals with TB aged 18 and above, including both males and females, who exhibit low treatment self-efficacy. The evaluation will assess the intervention's effects on treatment self-efficacy (primary outcome and secondary outcomes), including treatment adherence, intermediate mental and physical health outcomes, and infection control practices.

In accordance with a hybrid effectiveness-implementation design, longitudinal qualitative data will also be collected to triangulate with quantitative findings, thereby providing deeper insight into intervention mechanisms and contextual factors. The enhanced standard of care, comprising brief counseling and information, education, and communication (IEC) activities, will be compared to evaluate the added benefit of the self-efficacy-based intervention.

## Sample size

We estimated that our proposed intervention would reduce the proportion of individuals with poor self-efficacy from 50 to 35%, corresponding to an absolute difference of 15%. A sample size of 166 TB patients per arm (332 in total) was calculated assuming an average cluster size of 50, a significance level of α = 5%, β = 20% and a power 1–β = 80%.

**Figure 1:**
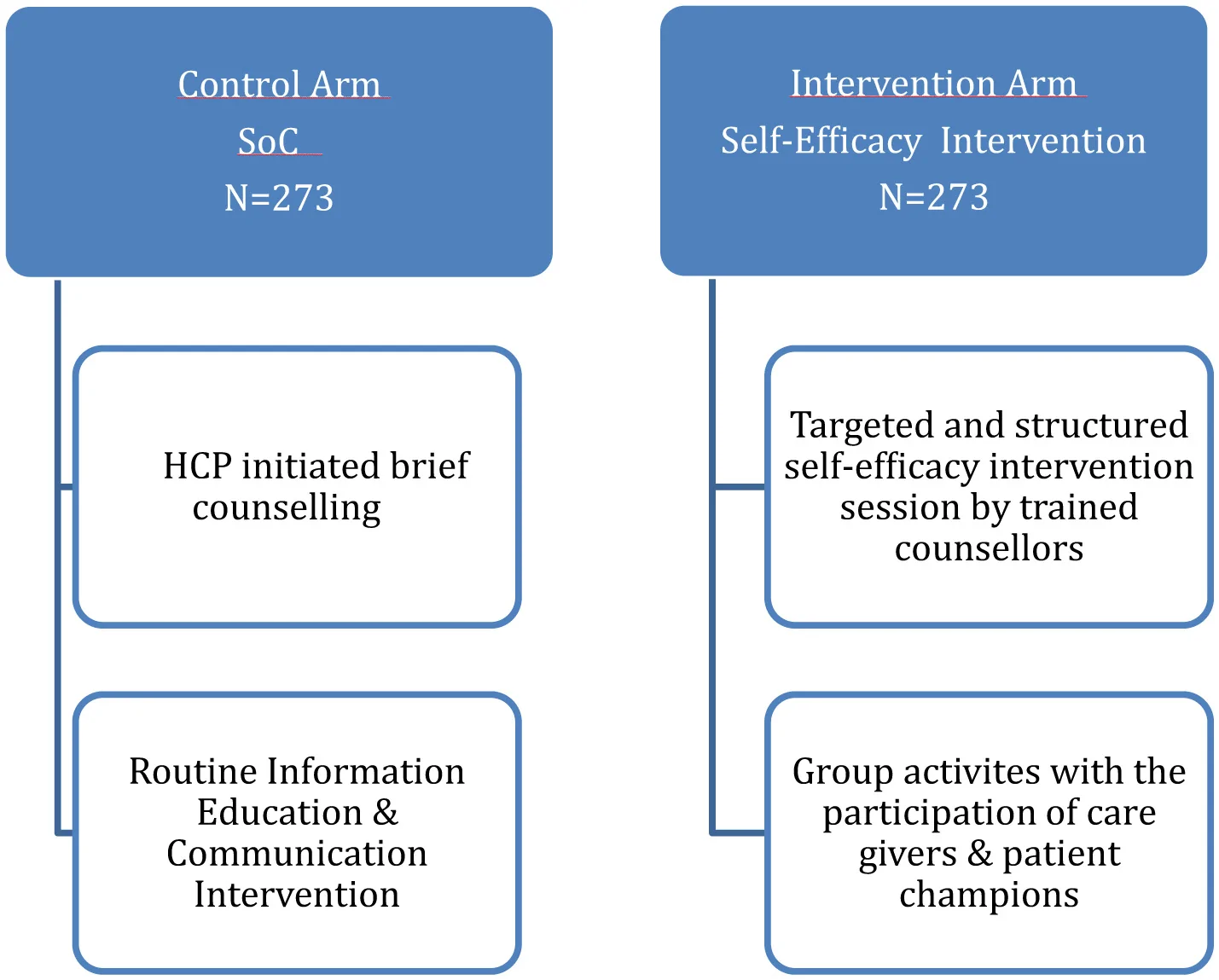
Study arm description.

Considering an average cluster size of 50, yielding a design effect of 1.5, we predict that we would require 500 participants, with five clusters in the control arm and five in the intervention arm. Accounting for an anticipated 10% dropout rate, we plan to screen and include approximately 547 participants overall. Arm wise sample size has been explained in Figure 1.

The sample size is calculated using the formula:

S = (zα + zβ) ∧ 2 ^*^ (C^*^(1–C) + I^*^(1–I)) / (C–I) ∧ 2, where C represents the expected proportion in the control arm and I represents the expected proportion of the intervention arms.

Participants will be screened based on their baseline self-efficacy score, and TB treatment facilities will be regarded as clusters. During the recruitment phase, the matching of average self-efficacy scores between the intervention and control clusters will be assessed. Recruitment within each cluster will continue until the required sample size per cluster is achieved. Eligible participants will be enrolled sequentially from the health centres within the designated selected TU.

## Intervention description

### Self-efficacy driven intervention

The intervention will comprise the following core components: motivational approach, behavioral skills-building and adaptations, self-management skills, and supportive care for patients and their caregivers. Each component will include specific, structured activities aligned with the principles of self-efficacy.

The intervention will be implemented through a combination of individual and group sessions that incorporate elements of cognitive behavioral therapy, individual counseling, and group therapy. The content of these sessions will include elements such as motivation, goal setting, psychoeducation, problem-solving, mind diversions, normalization, behavioral activation, and cognitive coping.

The intervention will be synchronized with the NTEP treatment schedule and integrated within the routine care practices. The intervention will be delivered across 10–12 structured sessions, beginning at treatment initiation and continuing through completion.

Session frequency and duration will vary according to the treatment phase:

- Initial phase (1st month): brief sessions of 10–15 min, delivered weekly.
- Intensive phase: sessions extended to 20–40 min, delivered fortnightly.
- Continuation/final phase: sessions maintained at 20–40 min, delivered monthly.

**Figure 2:**
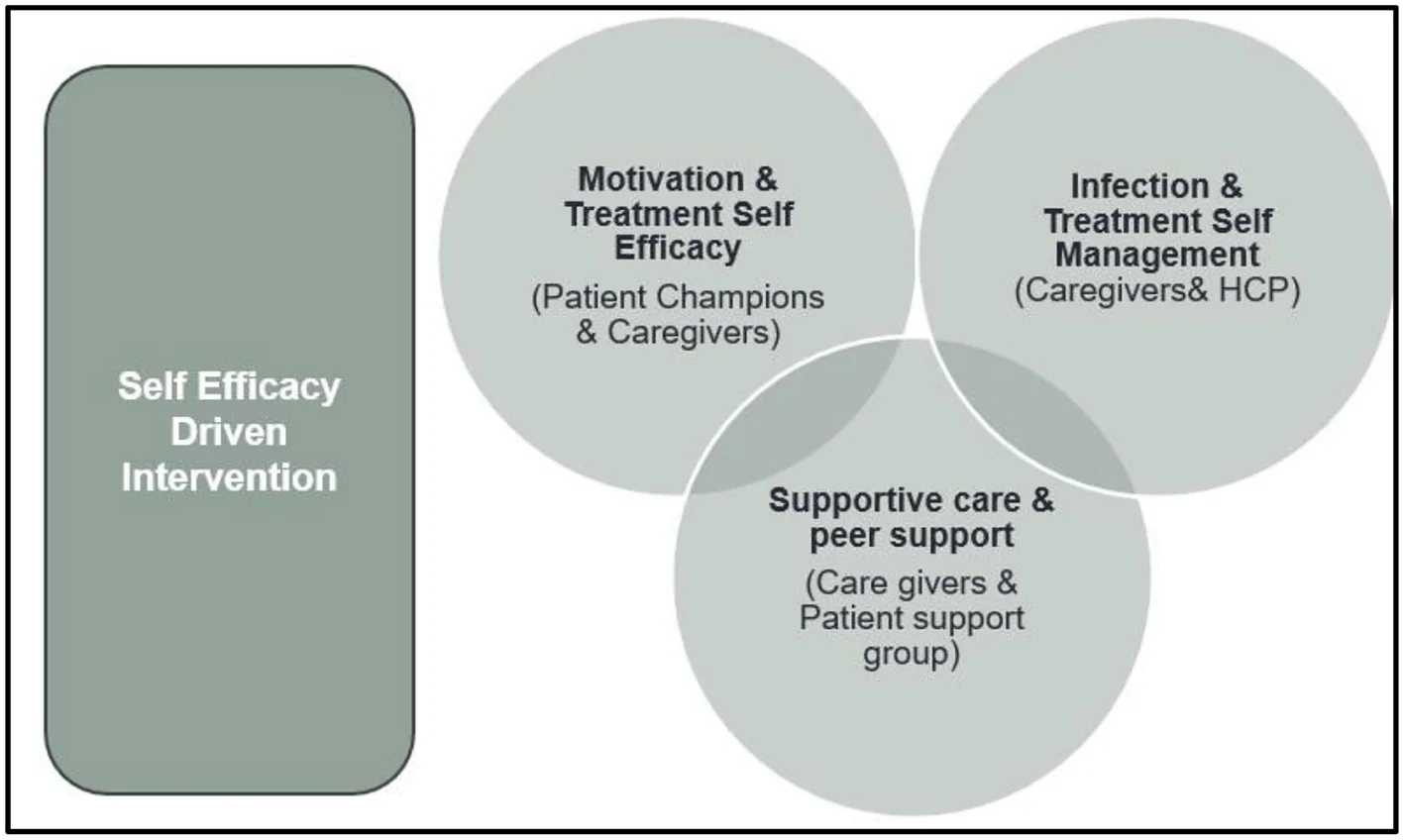
Components of an integrated self-efficacy driven intervention for individuals with TB and caregivers.

Interventions will take place at the TUs or local treatment centres where patients receive their care, with the option of home-based delivery for participants who prefer it. Each component of the intervention (Figure 2) will be administered by trained counselors by following a co-creation and adaptation framework (11, 13).

### Enhanced standard of care arm intervention

In accordance with the Declaration of Helsinki, the participants in the control arms of randomized trials, will receive highest available standard of care. Participants in the intervention arm will receive a brief counselling and motivational discussion in addition to the routine care provided by a frontline healthcare staff in TB health facilities.

Information, Education, and Communication (IEC) materials will be distributed to patients and caregivers, covering topics such as nutrition, risk behavior reduction, and infection control practices. Patients diagnosed with alcohol use disorder or depression will be referred to government psychiatric services for further evaluation, care, and follow-up.

## Evaluation and analysis: quantitative component

Healthcare centres within the selected TUs at the study sites will serve as recruitment points for eligible individuals on DS TB or DR TB treatment with oral shorter regimens, along with their caregivers.

Data collection will occur at three time points: treatment initiation, midpoint, and completion. At each stage, trained study personnel will administer the standardized questionnaires to participants in both the control and intervention arms. These questionnaires will cover a variety of topics, including patient self-efficacy related to TB treatment, medication adherence and infection control practices, TB-related stigma, depression, caregivers' self-efficacy, and stigma. Additionally, the air quality assessments will be conducted at the baseline and midpoint of treatment in the homes of selected and consenting patients using aerosol samplers to evaluate infection control conditions.

## Analysis of study outcomes data

The following standardized tools and scales will be used for the present study at three different time points: 1. Baseline (Initial phase of treatment), 2. Midline (3rd month of treatment), and 3. Endline (6th month of treatment), and on average, it takes 40–50 min to complete all the scales.

- Self-Efficacy for Managing Chronic Disease 6-item Scale (12).
- Caregivers' Self-Efficacy Scale (CSES-8) (14).
- TB Stigma Scale (15).
- Patient Health Questionnaire-9 (16).
- AUDIT-C scale for alcohol dependence (17).
- Airborne Infection Control (AIC) practices Survey Questionnaire (18).
- TB Medication adherence and Treatment outcomes (assessed at midline and endline).

All analyses will be conducted following an intent-to-treat (ITT) approach. Continuous outcomes, including self-efficacy score and other secondary outcomes (e.g., PHQ-9 scores, AUDIT-C scores, and TB stigma scores), will be analyzed using linear mixed models. Fixed effects in the model include:

- Treatment arm (0 = usual care; 1 = self-efficacy-based intervention).
- Time (0 = baseline, 1 = 3 months post-baseline; 2 = 6 months post-baseline).
- Interaction terms between the treatment arm and time (Treatment × Time interaction term).

This modeling approach will allow estimation of mean differences in change scores between study arms with 95% confidence intervals (CIs). Cohen's *D* will be calculated to estimate the effect size. Covariates showing meaningful baseline differences will be adjusted for in the models. For non-normally distributed continuous outcomes, generalized linear mixed effects models with the appropriate link functions will be employed.

## Analysis of intervention uptake and completion

To identify the predictors of intervention completion, a logistic regression model (logit model) with a binary outcome will be estimated, where:

- Outcome variable: completion status (1 = completer; 0 = non-completer).
- Predictor variables: demographic, clinical, and socio-economic factors.

Results will be reported as odds ratios (ORs) with corresponding 95% confidence intervals.

## Mediator/moderator analysis

To understand the direct and indirect effects of the self-efficacy-based intervention on TB-related stigma, medication adherence, and infection control practices, we will examine the potential mediating role of depression, alcohol use, and smoking using a counterfactual approach. By incorporating interaction terms into the previously specified models, we will also assess potential moderating effects of self-efficacy on other mental and behavioral health outcomes in addition to the main intervention effects.

## Qualitative component

A longitudinal qualitative research (LQR) design with a comparative approach will be implemented. This design will involve follow-up of patients and caregivers receiving either the self-efficacy-based intervention or enhanced standard care. Two focus group discussions (FGDs) will be conducted in each arm at two time points: treatment initiation and post-intensive phase, at each study site, utilizing the same cohort of participants. Purposive sampling will be employed to ensure diversity in participant experiences.

This qualitative component aims to gain an in-depth understanding of the psychosocial dynamics, coping mechanisms, and behavioral adaptations among TB patients who receive the intervention during their treatment, compared to those receiving enhanced standard care. Key domains explored in FGDs will include anxiety, stigma, discrimination, low self-esteem, hopelessness, physical incapacity, and a lack of motivation.

Additional topics will include awareness of TB symptoms, accessibility of treatment facilities, TB testing, treatment and preventive therapy, knowledge regarding TB prevention and execution of infection control measures (e.g., respiratory hygiene, sputum disposal, and ventilation) at both personal and household levels, barriers to implementing these practices, and support provided to household members undergoing treatment. Experiences related to stigma and social support will also be probed.

To triangulate findings, data from aerosol sampling, as detected using an aerosol sample detection apparatus, will be used to verify participants' self-reported experiences and insights related to infection control practices within their households.

The qualitative analysis will follow a systematic and multi-step process comprising: familiarization with raw data, generation of a coding framework, verification of data saturation, identification and refinement of the main themes and sub-themes, review, and organization of findings into thematic metrices.

Initially, all interviews will be audio-recorded, field notes will be taken, and the transcriptions will be made from the native language. Additionally, the quality of the transcription will be assessed, and the transcripts will be translated and validated in English to ensure the quality and accuracy. The data will be imported into NVivo 12 (QSR International) for a comprehensive thematic analysis to identify the initial broad themes. A framework matrix approach will be applied to analyse and compare the themes that reflect the patient's experiences and perspectives in both the intervention and control arms when juxtaposed. The differences and similarities in thematic analysis, reflecting motivation, self-efficacy, behavior adaptation, and infection control self-management, as well as the approach to medication adaptation, will be evaluated among patients in the two arms. Analyses will also account for variations by TB type, study site, and socio-demographic characteristics.

The study will adhere to the Consolidated Criteria for Reporting Qualitative Research (COREQ) checklist to ensure adherence to standards and rigor in qualitative data collection, analysis, and reporting. The qualitative studies published in the TB context by the National Institute for Research in Tuberculosis (NIRT) will serve as methodological references (19).

## Design for objective 3

A qualitative assessment will be conducted to collect data on the barriers and facilitators to implementing the self-efficacy-based intervention. Individual feedback interviews will be conducted with five TB patients and their caregivers (*n* = 5) per site in the intervention arm, representing diverse age and gender groups, who either completed or did not complete the self-efficacy-based intervention. In total, 20 interviews are planned per site, for a total of 40 across both sites. The participants will be selected purposively to ensure diversity.

Additionally, focus group discussions (FGDs) will be conducted by interventionists, NTEP care providers, and program-level stakeholders. Two FGDs will be conducted at each site, totalling four FGDs across both locations.

The qualitative inquiry will explore key implementation outcomes, such as acceptability, accessibility, feasibility, and sustainability. Themes related to barriers and facilitators influencing the effectiveness, adoption, and scalability of implementation will also be identified.

## Discussion

The ability of individuals with TB and their caregivers to overcome the multiple challenges they encounter is strongly influenced by their personal level of self-efficacy and motivation (11, 13). Self-efficacy is a central concept of Bandura's health belief model, which underpins an individual's capacity to engage in health behaviors, minimize risk behaviors, manage emotional distress, and develop self-management skills to consistently address treatment-related challenges and evolving needs.

The proposed self-efficacy-based intervention, if proven efficacious, will represent the first integrated, theory-driven, and evidence-based psycho-social intervention for TB patients in India. It has the potential to positively influence treatment acceptance and uptake, strengthen self-care behaviors, and holistically address underlying psychosocial issues. Evidence generated from this study could support the scaling up of self-efficacy interventions under the NTEP in a pragmatic and sustainable manner. This thereby improves treatment services and outcomes for TB patients nationwide.

We anticipate that this study will yield substantial benefits for the participants by providing critical insights into feasible, holistic psycho-social interventions that address low treatment self-efficacy, poor treatment adherence, and mental health challenges among TB patients. Such findings may lead to advances in care that could eventually improve/strengthen/enhance treatment outcomes for TB patients not only in India but also across other low- and middle-income countries facing similar challenges.

Furthermore, this study will provide valuable evidence demonstrating that targeted interventions at the patient and caregiver levels can improve infection control self-management, which is imperative for reducing TB transmission. The study will also contribute to understanding how family caregivers, as active participants in care, can play a vital role in supporting treatment adherence and infection prevention.

If proven effective, the tested interventions could be scaled up nationally to bridge gaps in patients' and caregivers' ability to overcome challenges related to TB treatment. Such an approach would directly contribute to achieving the 92% treatment completion target set by the NTEP and strengthen infection prevention practices at the household level, thereby reducing TB transmission among household contacts.
